# Urinary incontinence in women: prevalence rates, risk factors and impact on quality of life

**DOI:** 10.12669/pjms.293.3404

**Published:** 2013

**Authors:** Nazli Sensoy, Nurhan Dogan, Burcu Ozek, Leyla Karaaslan

**Affiliations:** 1 Nazli Sensoy, Department of Family Medicine, Afyon Kocatepe University, Faculty of Medicine, Afyonkarahisar, Turkey.; 2 Nurhan Dogan, Department of Biostatistics, Afyon Kocatepe University, Faculty of Medicine, Afyonkarahisar, Turkey.; 3 Burcu Ozek, 6th Grade Medical Student, Afyon Kocatepe University, Faculty of Medicine, Afyonkarahisar, Turkey.; 4 Leyla Karaaslan, 6th Grade Medical Student, Afyon Kocatepe University, Faculty of Medicine, Afyonkarahisar, Turkey.

**Keywords:** Awareness, Female, Prevalence, Risk factors, Urinary incontinence

## Abstract

***Objectives: ***To determine the prevalence, risk factors of urinary incontinence (UI) and to assess its impact on the quality of women’s life.

***Methods: ***This cross-sectional study was performed 1050 female participants aged between 20-80 years. A questionnaire form, including the socio-demographic characteristics and risk factors and the "International Consultation on Incontinence Questionnaire-Short Form" were used for the data collection.

***Results: ***The mean age of women was 48.80±11.53 years. The prevalence of UI was 44.6%. The distribution of the types of UI was 31% stress incontinence, 47.4% urge, and 33.1% mixed type. Although 95.5% of the women reported a negative impact on the quality of life, admission to a health center was only 63.9%, and 64.7% of the women had not received any medical help. The statistical analysis revealed that menopause, constipation, hypertension, diabetes, family history and parity are associated with UI as risk factors.

***Conclusion: ***We suggest that in the early diagnosis and treatment of urinary incontinence (UI), mental, educational and psychosocial support should be given to patients together with medical therapy.

## INTRODUCTION

Urinary incontinence (UI) has been described as “involuntary leakage of urine at an inappropriate time point and in an inappropriate place”.^1^ The types of UI have been designated as stress, urge, mixed, nocturnal, continuous type and others.^1^

Epidemiological studies conducted on UI show that the condition is 2-3 times more common in women^2^, UI can be considered as a normal part of aging when in fact it's not and is observed in women at any age group from different cultures and races, contrary to general opinion which is more common in elderly population, thus constituting a worldwide problem.^2^^-^^4^ In a systematic literature review, UI has been reported to have a wide prevalence interval with a rate of 16.2% to 81.9%.^5^ In studies conducted in Turkey, the prevalence of UI among women has been reported as 20.5-68.8%.^2^

Urinary incontinence is regarded as a disgraceful situation, with a negative effect on quality of life (QOL) and is usually kept disguised; it is an important disease leading to physical, social, psychological, sexual and economical problems among women of all age groups.^6^ The detection of the factors which cause UI and early diagnosis are the crucial for better protection and healing. The aim of this study was to determine the prevalence and associated risk factors of UI, to evaluate the effects on the quality of woman’s life, and awareness of women.

## METHODS

***Study design: ***A cross-sectional study was conducted on 1050 female patients aged from 20 to 80 year-old and referring to the outpatient clinics of Kocatepe University Hospital between December 2009 and January 2010.

***Data collection: ***The data were collected using a questionnaire prepared by the investigator and the Turkish version of the International Consultation on Incontinence Questionnaire Short Form (ICIQ-SF). The reliability and validity studies of the ICIQ-SF form were reported by Cetinel et al.^7^ The ICIQ-SF questionnaire which investigates the type, rate and severity of UI, as well as the impact on QOL is short, comprehensible and easy-to-apply.^8^ In the first section of the questionnaire participants were expected to give information related to their socio-demographic characteristics, risk factors for incontinence, obstetric history, habits, awareness status, namely the attitude towards this disturbing situation, presenting to a healthcare center (if any) and treatment for this condition (if any). In the second section, the ICIQ-SF was requested to be completed by each patient who gave a positive reply for the question “Did you have any complaint of UI in the last 4 weeks?”. The questions in the ICIQ- SF were to identify the types and severity of UI.

***Ethical considerations: ***Before conducting the survey, permission was obtained from the projects committee of Afyon Kocatepe University. Then the participants were informed about the study objectives and individual verbal informed consent was obtained according to the principles of the Declaration of Helsinki. The participants were then requested to complete the questionnaire. The questionnaire was completed by the investigators through a face-to face interview method for only the illiterate participants.

***Data analysis: ***The comparisons of prevalence between the dichotomous categories were made using the chi-square tests. The continuous variables were expressed as mean, percentage value and frequency. The epidemiological data were analyzed with the binary logistic regression models to evaluate the possible risk factors of UI. In the logistic regression, UI (positive or negative) was the dependent variable, while menopause, constipation, hypertension, diabetes mellitus (DM), family history of urinary leakage and the number of deliveries were independent variables. The Backward Stepwise (Wald) elimination of all the non-significant variables were applied to obtain a minimal model containing only significant variables. The odds ratios (OR) and the 95% Confidence Interval (CI) were estimated. All analyses were performed using the SPSS for Windows, version 18.0 and a *p *value of <0.05 was considered statistically significant.

## RESULTS

The mean age of the 1050 female participants was 48.80±11.53 (20-80); 82.4% of the participants (n=865) were married, 54% (n=567) were primary school graduates, 89.8% (n=943) were housewives and 34.5% (n=362) were obese. In the comparison of women with and without UI, significant differences were found in terms of socio-demographic characteristics (p<0.001 for all). The distribution of socio-demographic characteristics for these two groups was given in Table-I.

Urinary incontinence was found in 44.6% (n=468) of all cases. The symptoms of UI were classified according to the ICIQ-SF. Accordingly, the distribution of the types of incontinence was as follows: 47.4% (n=222) women had urge UI (UUI), 31% (n=145) had stress UI (SUI) and 33.1% (n=155) had mixed UI (MUI). The frequency of UI in once a day or more was 52.8% of cases. A trace amount of UI was found in 66.2%. Among women replying the question in the ICIQ-SF regarding “what is the impact of UI on your daily life”, the rate of reporting a negative impact on QOL was 95.5% (Table-II).

It was also determined that while 57.1% of women with UI (n=267) regarded this condition as a “health problem”, 63.9% (n=299) did not admit to a healthcare center and 64.7% (n=303) did not receive any medical help for UI. The reasons for not admitting to a center for UI were regarding the condition as a natural consequence of advanced age (28.8%), feeling humiliated of being examined (20.1%), no time for medical examination (16.7%), and not feeling uncomfortable UI (16.4%).

However, the assessment of the UI risk factors revealed significant differences between women with and without UI in terms of factors other than interventional delivery, smoking and history of radiotherapy (Table-III).

According to the binary logistic regression analysis, menopause (p<0.001), constipation (p<0.001), hypertension (p<0.013), DM (p<0.05), family history of UI (p<0.001) and the number of deliveries (p<0.05) were indicated as significant risk factors. The risk of UI was approximately three times higher in menopausal women compared to non-menopausal women, and the risk in individuals with a family history of UI was approximately two times higher than the others.

## DISCUSSION

Urinary incontinence is a common problem among women in all age groups.^4^ The rate of prevalence varies in different countries. In some studies, while the interval of prevalence rates were given as 10-51%, the corresponding rates in stress, urge and mixed type UI were indicated as 22.9-57%, 2.8-23% and 12.4-51.4%, respectively.^4^^,^^9^^-^^13^ The wide range of prevalence rates in these studies may be related with utilization of various definitions, study groups, type of the study, response rate, age, gender, availability and efficiency of healthcare and other factors.^2^^,^^4^ In our study, the prevalence of UI in women was 44.6% and the rates of SUI and MUI were 31%, 33.1%, respectively which were equal to the ranges reported in previous studies. However, the rate of UUI (47.4%) was approximately two times higher than the rates of previous studies.

Several studies have reported the association of age and gender with prevalence of UI.^7^^,^^9^^-^^12^^,^^14^ In a study, the risk of SUI was found to be approximately 6 times more common in individuals over the age 40, while the risk of UUI was two times higher in the corresponding group.^15^ This study detected that the rate of UI increased with advanced age, especially between 40-49 years.

**Table-I:** Socio-demographic characteristics of participants

	*Urinary Incontinence*		
*Characteristics*	*No n (%)*	*Yes n (%)*	*p*
Age-group (years)			
20-39	181 (31.1)	54 (11.5)	<0.001
40-49	209 (35.9)	150 (32.1)	
50-59	126 (21.6)	127 (27.1)	
≥ 60	66 (11.3)	137 (29.3)	
*Marital status*			
Married	504 (86.6)	361 (77.1)	<0.001
Single	23 (4.0)	6 (1.3)	
Divorced/Widow	55 (9.5)	101 (21.6)	
*Education*			
Literate	120 (20.6)	185 (39.5)	<0.001
Primary/Secondary	335 (57.6)	232 (49.6)	
High/University	127 (21.8)	51 (10.9)	
*Occupation*			
Housewife	508 (87.3)	435 (92.9)	<0.001
Retired	14 (2.4)	10 (2.1)	
Working	60 (10.3)	23 (4.9)	
*Body mass index, kg/m* ^2^			
18.5-24.9	144 (24.7)	100 (21.4)	<0.001
25-29.9	266 (45.7)	178 (38.0)	
≥ 30	172 (29.6)	190 (40.6)	

**Table-II:** Frequency, impact on the quality of life, and amount of leakage in women with urinary incontinence

*Characteristics of UI (n=468)*	*n*	*%*
*Frequency of leakage*		
Once a week or less	112	23.9
Twice or three times a week	109	23.3
Once a day	72	15.4
Few times a day	99	21.2
Always	76	16.2
*Amount of leakage*		
Small	310	66.2
Moderate	82	17.5
Large	76	16.2
*Impact on QoL*		
0: not at all	21	4.5
1-3: mild	170	36.3
4-6: modarete	134	28.6
7-9: severe	126	26.9
10: great extent	17	3.6

**Table-III:** Comparison of potential risk factors between women with and without UI

*Risk Factors*	*Incontinence Yes n (%)*	*Incontinence No n (%)*	*p*
*Number of Deliveries*			
0 1 – 2 ≥3	23 (34.3) 123 (35.4) 322 (50.6)	44 (65.7) 224 (64.6) 314 (49.4)	<0.01
*Episiotomy*			
No Yes	410 (46.3) 58 (35.2)	475 (53.7) 107 (64.8)	<0.01
*Interventional Delivery*			
No Yes	439 (44.8) 29 (42.0)	542 (55.2) 40 (58.0)	0.66
*Miscarriage*			
Yes No	194 (52.6) 259 (41.4)	175 (47.4) 366 (58.6)	<0.01
*Abortion*			
No Yes	289 (42.0) 161 (53.1)	399 (58.0) 142 (46.9)	<0.01
*Age at first delivery (years)*			
≤ 20 21-25 ≥26	319 (49.2) 113 (40.5) 17 (28.8)	330 (50.8) 166 (59.5) 42 (71.2)	<0.01
*4 kg baby delivered*			
No Yes	313 (43.4) 138 (51.7)	409 (56.6) 129 (48.3)	<0.01
*Menopause*			
No Yes	163 (29.7) 305 (60.9)	386 (70.3) 196 (39.1)	<0.01
*Smoking*			
No Yes Quit	397 (45.1) 41 (37.3) 30 (50.8)	484 (54.9) 69 (62.7) 29 (49.2)	0.183
*Coffee drinking*			
No Yes	340 (49.5) 128 (35.3)	347 (50.5) 235 (64.7)	<0.01
*Urinary tract enfection*			
None 1 or 2 times per year 3 times or over per year	190 (39.3) 164 (47.1) 114 (52.3)	294 (60.7) 184 (52.9) 104 (47.7)	<0.01
* Constipation*			
No Yes	220 (37.2) 248 (54.1)	372 (62.8) 210 (45.9)	<0.01
*Cough*			
No Yes	399 (43.3) 69 (53.9)	523 (56.7) 59 (46.1)	<0.05
*Hysterectomy*			
No Yes	393 (42.8) 75 (56.8)	525 (57.2) 57 (43.2)	<0.01
*Radiotherapy*			
No Yes	461 (44.5) 7 (53.8)	576 (55.5) 6 (46.2)	0.49
*Family history of incontinence*			
No Yes	352 (42.6) 116 (52.0)	475 (57.4) 107 (48.0)	<0.05

Different studies showed that the women with UI were determined to be affected more frequently, due to obstetric, gynecological and hormonal causes^2^; changes in urinary bladder and pelvic structures, chronic disease and menopausal stages were generally suggested as correlative factors.^12^^,^^16^

In the type and severity of UI such as amount, frequency and duration, the weight, stress, help-seeking behavior and age were reported as significant variables with an impact on QOL. ^4^^,^^5^. The rate of patients reporting UI once daily or more was found 63.2% in a study but 42.1% in another study.^6^^,^^11^ In the same studies the rates of patients reporting a trace amount of UI in those studies were determined as 79.8%, 78.7%, respectively.^6^^,^^11^ In our study, while it was determined that the rate of patients reporting UI once daily or more was 52.8%, the rate of cases with a trace amount of UI was 66.2%.

Different studies have reported that many of the women with UI were not receiving any medical help despite the presence of a negative impact on QOL.^4^^,^^6^^,^^11^ Similarly, we found that 63.9% of women with UI did not admit to a center and 64.7% did not receive any medical help, although 95.5% described this problem as a negative impact on QOL and 57.1% accepted it a disease. In some studies, their overall causes of not seeking medical care were indicated as: cultural and ethnic diversities, regarding UI as a social problem and as a taboo issue, considering UI as a normal component of the aging process, low expectancy in terms of benefits of treatment, being humiliated, the type and severity of UI and impact on QOL, indecisiveness, lack of knowledge regarding where to present for treatment, difficulty and fear in consulting health professionals and relatively high cost of diagnosis, treatment and consultancy services.^2^^,^^4^^,^^17^ Our study determined that their causes of not seeking medical care were indicated as advanced age, feeling humiliated of being examined, no time for medical examination and no discomfort due to UI. These results suggest that in general, the women with UI did not consider this condition as a life-threatening problem and did not have information about available treatment methods.

In many studies, the factors associated with UI have been indicated as age, female gender, level of education, marital status, obesity, number of deliveries, abortion, age at first delivery, hysterectomy, menopause, history of urinary infection, constipation, coughing, DM and hypertension.^4^^,^^6^^,^^9^^-^^13^ At the same time, in several studies, smoking, delivery of an infant with a weight of 4 kg or over and episiotomy were reported to have an impact on UI, while in several other studies, these factors were indicated as ineffective.^2^^,^^4^^,^^10^^,^^12^^,^^13^^,^^15^ In our study, a significant correlation was found between UI and some of these risk factors such as menopause, constipation, hypertension, DM, family history of UI and the number of deliveries in the Binary Logistic Regression analysis.

Urinary incontinence is an important multifactorial health problem which affects women’s life quality negatively and related significantly to age, education, occupation, marital status and BMI. However, because of social, psychological and cultural problems, many of women with UI do not admit to health center to get support. Therefore, healthcare professionals should concentrate their efforts on improving the awareness of this problem among women and assure them that this is not a condition of humiliation and taboo while providing appropriate medical care, simple lifestyle changes, and psychosocial support.
